# Пангипопитуитаризм как первое проявление генерализованного саркоидоза: клинический случай

**DOI:** 10.14341/probl13115

**Published:** 2022-06-15

**Authors:** Ю. А. Уханова, И. А. Иловайская, С. А. Терпигорев

**Affiliations:** Московский областной научно-исследовательский клинический институт им. М.Ф. Владимирского; Московский областной научно-исследовательский клинический институт им. М.Ф. Владимирского; Московский областной научно-исследовательский клинический институт им. М.Ф. Владимирского

**Keywords:** пангипопитуитаризм, саркоидоз, нейросаркоидоз, саркоидная гранулема, клинический случай

## Abstract

Саркоидоз — это системное воспалительное заболевание неизвестной этиологии, характеризующееся образованием неказеифицирующихся гранулем в различных органах и тканях и активацией Т-клеток в месте гранулематозного воспаления с высвобождением различных хемокинов и цитокинов. Заболеваемость в среднем составляет от 10 до 20 на 100 000 населения. Наиболее часто у больных саркоидозом выявляют поражение легких и внутригрудных лимфатических узлов. Существенно реже (примерно у 5–20% пациентов) отмечается поражение нервной системы. У 9–18% пациентов с нейросаркоидозом обнаруживают вовлечение гипофиза, воронки гипофиза и гипоталамуса, что проявляется разнообразной клинической симптоматикой. Мы наблюдали больного саркоидозом, у которого заболевание дебютировало с клинических симптомов гипогонадизма с последующим развитием признаков вторичного гипотиреоза, надпочечниковой недостаточности и несахарного диабета, что поначалу расценивалось как пангипопитуитаризм на фоне поражения гипоталамуса неясного генеза. Через некоторое время при дообследовании выявлены признаки внутригрудной лимфаденопатии и очаговые изменения в легочной паренхиме на КТ, а также поражение кожи. Несмотря на биохимическую компенсацию гипопитуитаризма, клиническая эффективность гормональной терапии препаратами каберголина, тестостерона, гидрокортизона и левотироксина натрия оказалась недостаточной, а улучшение состояния пациента произошло после присоединения иммуносупрессивной и противовоспалительной терапии метотрексатом и метилпреднизолоном.

## Abstract (en)

Sarcoidosis is a systemic inflammatory disease of unknown etiology characterized by the formation of noncaseating granulomas in various organs and tissues and the activation of T-cells at the site of granulomatous inflammation with the release of various chemokines and cytokines [1]. The incidence on average ranges from 10 to 20 per 100,000 population [2]. Most often in patients with sarcoidosis, lesions of the lungs and intrathoracic lymph nodes are detected. Significantly less often (in about 5–20% of patients) damage to the nervous system is noted [6,7,9]. In 9-18% of patients with neurosarcoidosis, involvement of the pituitary gland, pituitary infundibulum and hypothalamus is found, which is manifested by a variety of clinical symptoms [8,10]. We observed a patient with sarcoidosis whose disease debuted with clinical symptoms of hypogonadism, followed by the development of signs of secondary hypothyroidism, adrenal insufficiency, and diabetes insipidus, which was initially regarded as panhypopituitarism against the background of a hypothalamic lesion of unknown origin. Later, additional examination revealed signs of intrathoracic lymphadenopathy and focal changes in the lung parenchyma on CT, as well as skin lesions. Despite the biochemical compensation of hypopituitarism, the clinical efficacy of hormonal therapy with cabergoline, testosterone, hydrocortisone and levothyroxine sodium was insufficient, and the patient’s condition improved after the addition of immunosuppressive and anti-inflammatory therapy with methotrexate and methylprednisolone.

## АКТУАЛЬНОСТЬ

Саркоидоз — это системное воспалительное заболевание неизвестной этиологии, характеризующееся образованием неказеифицирующихся гранулем, мультисистемным поражением различных органов и активацией Т-клеток в месте гранулематозного воспаления с высвобождением различных хемокинов и цитокинов [1]. Заболеваемость варьирует во всем мире и в среднем составляет от 10 до 20 на 100 000 населения [2]. Чаще страдают взрослые от 20 до 40 лет [3]. Распространенность саркоидоза среди женщин и мужчин примерно одинакова [4][5]. Наиболее часто при саркоидозе поражаются легкие, кожа и лимфатические узлы, однако у 5–20% пациентов может отмечаться вовлечение в патологический процесс нервной системы — так называемый нейросаркоидоз с соответствующими неврологическими симптомами [6][7][9].

При нейросаркоидозе наиболее часто поражаются черепно-мозговые нервы (до 21% случаев). Поражение гипофиза, воронки гипофиза и гипоталамуса наблюдается крайне редко – всего лишь у 9–18% ­пациентов с нейросаркоидозом [8][10]. Поданным систематического обзора и метаанализа, выполненных D. Fritz et al. за период с 1980 по 2016 гг., нейроэндокринные нарушения встречались в 9% всех случаев саркоидоза и включали пангипопитуитаризм, несахарный диабет, аменорею игалакторею у женщин. У большинства пациентов неврологические симптомы развиваются на фоне уже диагностированного «классического» саркоидоза [9]. Мы представляем клинический случай генерализованного саркоидоза, который дебютировал с нейроэндокринных симптомов вследствие формирования саркоидной гранулемы в гипоталамической области.

## ОПИСАНИЕ СЛУЧАЯ

Пациент М. в возрасте 25 лет (с 2005 г.) стал отмечать уменьшение оволосения на лице (стал бриться 1 раз в неделю). Постепенно стало снижаться либидо, беспокоили эректильная дисфункция, общая слабость.

В возрасте 27 лет (в июле 2007 г.) впервые обратился за медицинской помощью к эндокринологу. По результатам проведенного гормонального обследования выявлен гипогонадизм на фоне гиперпролактинемии (табл. 1). Выполнена мультиспиральная компьютерная томография головного мозга 04.07.2007: по заключению, данных за структурные изменения гипоталамо-гипофизарной области не получено. Состояние расценено как гиперпролактинемический гипогонадизм, назначен каберголин в дозе 0,5 мг 0,5 таблетки 2 раза в неделю. На этом фоне уровень пролактина нормализовался, однако значительного улучшения эректильной функции пациент не отметил, сохранялось снижение уровней тестостерона и гонадотропинов. Выполнена магнитно-резонансная томография (МРТ) головного мозга без контрастирования от 27.10.2007: гипофиз обычных размеров, гиподенсное образование размерами 2,5х4 мм, других образований в гипоталамо-гипофизарной области неописано. В связи с тем что в семье планировали беременность, дополнительно к приему каберголина был назначен препарат хорионического гонадотропина человека (ХГЧ) 2000 Ед 2 раза в неделю. На фоне данной терапии уровень тестостерона нормализовался, состояние пациента улучшилось.

**Table:** Таблица 1. Динамика лабораторных показателей пациента М. за время наблюденияTable 1. Dynamics of laboratory parameters of patient M. during the observation period *Примечание: ПРЛ — пролактин; ЛГ — лютеинизирующий гормон; ФСГ — фолликулостимулирующий гормон; ТТГ — тиреотропный гормон; Т4 св. — Т4 свободный; АКТГ — адренокортикотропный гормон; ИФР-1 — инсулиноподобный фактор роста 1 типа.

Лабораторные показатели
Год	ПРЛ, мМЕ/л(60–630)	ЛГ, МЕ/л(1,5–9,0)	ФСГ, МЕ/л(1–10)	Т общий, нмоль/л(9,0–38)	ТТГ, мкЕд/мл(0,2–4)	Т4 св., пмоль/л(11–23)	АКТГ 8.00, пг/мл(10–35)	Кортизол 8.00, нмоль/л(123–626)	ИРФ-1, нг/мл(177–382)
Июль, 2007	1063,71	1,01	2,1	1,0	2,42	17,1	-	-	-
Октябрь, 2007	500	1,4	1,03	7,44	1,3	13,2	-	-	-
2012	507	0,1	0,9	0,69	0,9	4,5	4,4	47	83
2017	138	0,6	0,9	0,69	-	10	-	-	-
2021	129,8	-	-	17,5	-	14,3	-	-	-

В период с 2008 по 2012 гг. к врачам не обращался, обследование и коррекция терапии не проводились. В ходе опроса выяснилось, что дозы препаратов ХГЧ и каберголина пациент самостоятельно снижал с последующей отменой в связи с плохим самочувствием в виде выраженной слабости и повышенной утомляемости.

В 32 года (в 2012 г.) обратился к эндокринологу в связи с нарастающей слабостью, снижением АД до 50/20 мм рт.ст., жалобами на снижение либидо и эректильную дисфункцию. При гормональном обследовании вновь выявлены умеренная гиперпролактинемия, вторичный гипогонадизм, а также диагностированы вторичный гипотиреоз, вторичная надпочечниковая недостаточность и сниженный уровень инсулиноподобного фактора роста 1 (табл. 1). По данным МРТ головного мозга с контрастированием от 05.04.2012: в проекции гипоталамуса, сосцевидных тел и прилежащих отделов воронки определяется образование размерами 9×11×12,5 мм с неровными четкими контурами, вертикальный размер гипофиза снижен до 4–5 мм. С полученными результатами был проконсультирован нейрохирургом: оперативное лечение невозможно в связи с особенностями локализации образования, показано консервативное лечение. Была назначена следующая медикаментозная терапия: каберголин 0,5 мг 0,5 таблетки 2 раза в неделю, препараты тестостерона 1000 мг в/м 1 раз в 12 нед (в связи с выраженным ухудшением общего состояния вопрос фертильности для пациента был неактуален), левотироксин натрия 100 мкг в сутки, преднизолон 10 мг в сутки. На фоне лечения пациент отметил улучшение самочувствия в виде стабилизации АД, улучшения эректильной функции, уменьшения общей слабости. По гормональным параметрам отмечалась нормализация уровней пролактина, тестостерона, Т4 свободного.

В 34 года (в 2014 г.) впервые появились жалобы на выраженную жажду (до 7 л в сутки), учащенное обильное мочеиспускание, сухость во рту, головную боль, сохранялась общая слабость. Выполнен анализ суточной мочи по Зимницкому, по результатам которого плотность мочи составила 1001–1003, общее количество мочи за сутки – 6 л. Выполнена МРТ головного мозга с контрастированием от 03.10.2014: образование в проекции гипоталамуса, сосцевидных тел и прилежащих отделов воронки увеличилось в размерах, «пустое» турецкое седло, характерной яркости МР-сигнала в области нейрогипофиза не ­отмечено (рис. 1). Уровни гликемии, калия и кальция в пределах референсных значений. Диагностирован несахарный диабет центрального генеза. Дополнительно к проводимой заместительной терапии назначен десмопрессин 0,2 мг 2 раза в сутки. На фоне скорректированного лечения жажда, учащенное мочеиспускание, сухость во рту не беспокоили, диурез менее 3 л, уменьшилась общая слабость.

**Figure:**
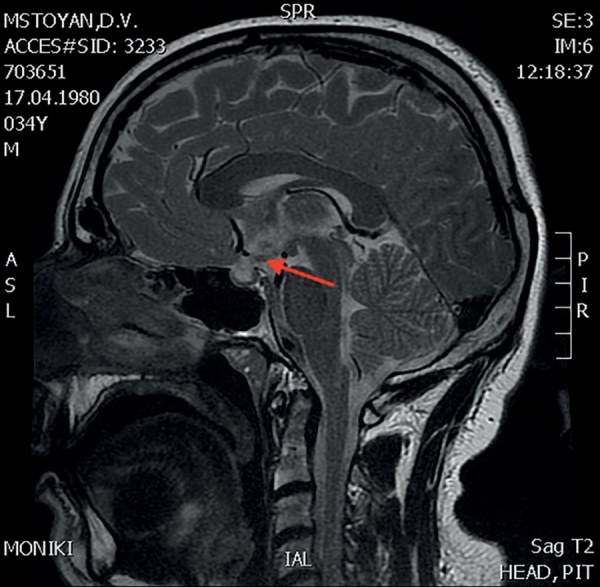
Рисунок 1. МРТ головного мозга с к/у от 03.10.2014г.(3,0 Тл)Саркоидная гранулема в проекции гипоталамуса, сосцевидных тел и прилежащих отделов воронки (указано стрелкой). Figure 1. MRI of the brain with c/c dated 03.10.2014 (3.0 T)Sarcoid granuloma in the projection of the hypothalamus, mastoid bodies and adjacent parts of the infundibulum (indicated by an arrow).

Далее периодически проводилось гормональное обследование в динамике, пангипопитуитаризм и несахарный диабет были скомпенсированы, однако жалобы на быструю утомляемость, выраженную слабость, головную боль прогрессировали.

В 36 лет (в 2016 г.) стали беспокоить кашель, одышка. При проведении флюорографии органов грудной клетки выявлены очаговые образования в легких. Диагноз туберкулеза был исключен. Пациент был консультирован пульмонологом. При активном расспросе выяснилось, что с 2008 г. пациент отмечал появление на коже бугорков фиолетового цвета, которые появлялись преимущественно при длительном пребывании на солнце, затем спонтанно регрессировали, пациент не обращал наних внимания и никогда не указывал при обращении к врачу. При КТ органов грудной клетки (рис. 3–5) в легочной паренхиме выявлены множественные мелкие очаги перилимфатического распределения, а также лимфаденопатия средостения. Данные изменения с учетом клинико-лабораторной картины заболевания не противоречили диагнозу «саркоидоз с поражением легких, внутригрудных лимфоузлов, кожи, гипоталамо-гипофизарной области». От проведения морфологической верификации диагноза пациент отказался, однако особенности течения заболевания позволили исключить альтернативные причины изменений на КТ и определили показания к противовоспалительной и иммуносупрессивной терапии. Была начата терапия метилпреднизолоном в дозе 24 мг в сутки и метотрексатом 5 мг 1 раз в неделю, которую пациент принимал в течение 6 мес. Затем доза метилпреднизолона была снижена до 8 мг в сутки. На фоне проводимого лечения состояние пациента улучшилось, по данным МРТ головного мозга в динамике (от 24.11.2017) ранее выявленное образование в гипоталамо-гипофизарной области не определялось, МР-картина «пустого» турецкого седла (рис. 2). В связи с выраженной слабостью на фоне основного заболевания пациент самостоятельно отменил препараты левотироксина натрия, тестостерона, десмопрессина и каберголина, продолжая только прием метилпреднизолона. Слабость усилилась, увеличилось потребление жидкости до 5 л в сутки. При очередном гормональном обследовании отмечалось отсутствие компенсации, было подтверждено наличие пангипопитуитаризма и центрального несахарного диабета, т.е. нейроэндокринные нарушения носили необратимый характер (табл. 2).

**Figure:**
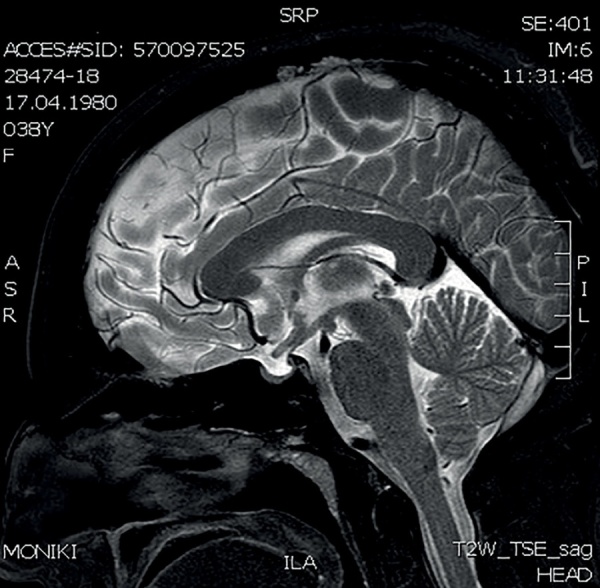
Рисунок 2. МРТ головного мозга с к/у от 24.11.2017г (3 Тл)МР-картина «пустого» турецкого седла, ранее выявленное образование в гипоталамо-гипофизарной области не определяется. Figure 2. MRI of the brain with an appointment dated November 24, 2017 (3 T)MRI picture of an “empty” sella turcica, a previously identified formation in the hypothalamic-pituitary region is not detected.

**Figure:**
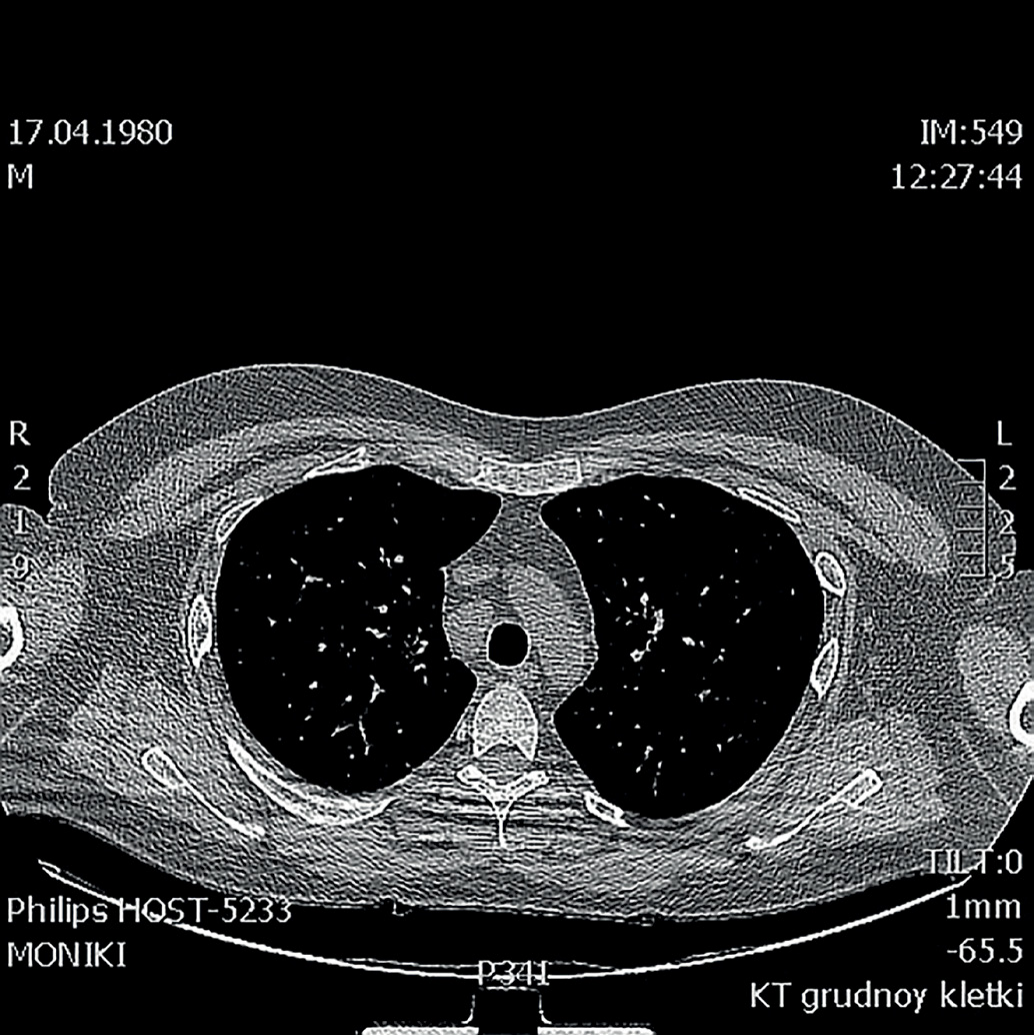
Рисунок 3. КТ органов грудной клетки от 2018г.(Немногочисленные мелкоочаговые тени лимфогенного распределения, лучше визуализируются в проекции междолевой плевры правого легкого). Figure 3. CT scan of the chest from 2018(A few small focal shadows of the lymphogenous distribution are better visualized in the projection of the interlobar pleura of the right lung).

**Figure:**
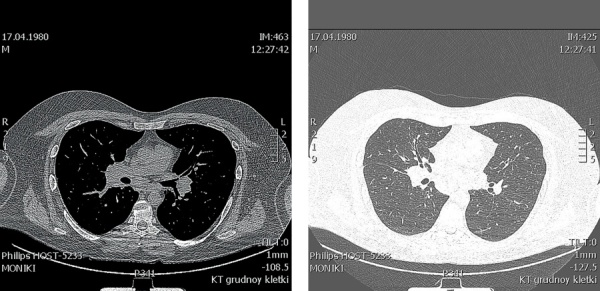
Рисунок 4, 5. КТ органов грудной клетки от 2018г.(Увеличение лимфоузлов средостения (паратрахеальных и бифуркационных)). Figure 4, 5. CT scan of the chest organs from 2018(Enlarged mediastinal lymph nodes (paratracheal and bifurcation))

**Table:** Таблица 2. Последовательность диагнозов, устанавливаемых в процессе наблюдения пациента М.Table 2. The sequence of diagnoses established during the observation of patient M *Примечание: гиперПРЛ — гиперпролактинемия; ГГ — гипогонадотропный гипогонадизм; ВГ — вторичный гипотиреоз; ВНН —вторичная надпочечниковая недостаточность; СТГ-недост-ть — СТГ-недостаточность; ЦНД — центральный несахарный диабет; С — саркоидоз.

год	гиперПРЛ	ГГ	ВГ	ВНН	СТГ-недост-ть	ЦНД	С
2007	+	+	-	-	-	-	-
2012	+	+	+	+	+	-	-
2014	+	+	+	+	+	+	-
2016	+	+	+	+	+	+	+

По данным КТ органов грудной клетки от мая 2020 г. отмечаются уменьшение размеров внутригрудных лимфоузлов, исчезновение очаговых затемнений в легочной паренхиме. В настоящее время пациент находится на терапии метилпреднизолоном вдозе 4 мг, а также на заместительной гормональной терапии каберголином в дозе 0,5 мг 0,5 таблетки 2 раза в неделю, препаратами тестостерона 1000 мг в/м 1 раз в 12 нед, левотироксина натрия в дозе 125 мкг в сутки и десмопрессина 0,2 мкг 2 разав сутки. На фоне данного лечения достигнута медикаментозная компенсация гипопитуитаризма. Клинических проявлений несахарного диабета в настоящее время нет.

## ОБСУЖДЕНИЕ

Ввиду разнообразия клинических проявлений нейросаркоидоза диагностика этой формы заболевания представляет определенные сложности. Подобные очаги поражения можно обнаружить, например, при туберкулезе, грибковой инфекции. J.P. Zajicek et al. предложили классификацию критериев для диагностики нейросаркоидоза, которая состоит из трех групп: 1) определенные; 2) вероятные; 3) возможные (табл. 3) [11][12].

**Table:** Таблица 3. Классификация диагностических критериев нейросаркоидоза по Zajicek J.P.Table 3. Classification of diagnostic criteria for neurosarcoidosis according to Zajicek J.P

Определенные	Клинические проявления, характерные для нейросаркоидоза.Данные гистологического исследования биоптата нервной ткани, подтверждающие диагноз нейросаркоидоза.Иключение других возможных диагнозов
Вероятные	Клинические проявления, характерные для нейросаркоидоза.Наличие признаков воспалительного процесса в ЦНС (повышение уровней белка, олигоклональных антител или лимфоцитарный плейоцитоз в спинномозговой жидкости, характерные изменения по результатам МРТ).Системный характер саркоидоза (гистологическое подтверждение, в том числе проба Квейма или наличие как минимум двух косвенных признаков из трех: сцинтиграфия с галлием, лимфаденопатия при визуализации грудной клетки, повышенная активность ангиотензин-превращающего фермента в сыворотке крови).Исключение других возможных диагнозов
Возможные	Клинические проявления, характерные для нейросаркоидоза.Исключение других возможных диагнозов, для которых характерны определенные и вероятные критерии нейросаркоидоза

Наиболее частыми проявлениями нейросаркоидоза при поражении гипоталамо-гипофизарной области являются несахарный диабет, гипогонадотропный гипогонадизм, гиперпролактинемия и вторичный гипотиреоз [8][12][13]. G. Murialdo и G. Tamagno проанализировали данные литературы с 1943 по 2001 гг. на предмет клинических проявлений при нейросаркоидозе у пациентов с поражением гипоталамо-гипофизарной области (всего 91 пациент), сопоставили с данными собственных клинических наблюдений и объединили результаты. Согласно этим данным, наиболее распространенным клиническим проявлением являлся гипогонадотропный гипогонадизм (38,5%), далее несахарный диабет (37,3%) [14]. В нашем клиническом случае нейросаркоидоз дебютировал с симптомов гипогонадизма и гиперпролактинемии, затем развились пангипопитуитаризм и несахарный диабет. Последовательность выпадения тропных функций гипофиза была сравнима с таковой, которая наблюдается при постепенном росте макроаденомы гипофиза [15]. Однако в данном случае постепенно увеличивалось образование в гипоталамической области, которое впоследствии оказалось саркоидной гранулемой.

Первой линией лечения при саркоидозе является иммуносупрессивная терапия, часто применяют длительный (не менее 6–12 мес) курс высокими дозами глюкокортикостероидов. Если на фоне лечения саркоидоза отмечается уменьшение, а иногда даже полное исчезновение образования, выявленного в тканях центральной нервной системы, это является подтверждением диагноза нейросаркоидоза и доказательством того, что данное образование было саркоидной гранулемой. Как известно, методом диагностики саркоидоза является морфологическое исследование пораженного органа (чаще всего — легкого и внутригрудного лимфоузла). Выявление в биоптате типичных саркоидных гранулем подтверждает предполагаемый диагноз. Однако такой стандартный диагностический подход на практике реализуется не всегда. В ряде случаев, например при типичном синдроме Лефгрена, проведение биопсии нецелесообразно, поскольку клинические проявления этого синдрома (лихорадка, узловатая эритема, артриты, увеличение внугригрудных лимфоузлов) высокоспецифичны для острой формы саркоидоза, а вероятность ошибочного диагноза меньше, чем риск осложнений биопсии. В случае саркоидоза хронического течения дифференциальный ряд включает несколько заболеваний (как правило — туберкулез, лимфому, грибковую инфекцию, бериллиоз), и при типичных изменениях КТ-картины (симметричное увеличение лимфоузлов корней легких, увеличение лимфоузлов средостения, мелкоочаговые тени лимфогенного распределения в легочной паренхиме), а также в отсутствие клинико-лабораторных признаков активности системного воспаления вероятность инфекции или лимфопролиферативного заболевания значимо снижается. При самопроизвольной положительной динамике КТ-картины вероятность альтернативных диагнозов весьма низкая. В приведенном клиническом примере пациент отказался от проведения биопсии, что обычно неявляется основанием для отказа от лечения в случае высоковероятного диагноза саркоидоза. В данном случае альтернативный диагноз маловероятен, учитывая КТ-картину типичного поражения легких и внутригрудных лимфоузлов, положительную динамику на фоне специфического для саркоидоза лечения, а также клиническую симптоматику поражения кожи и нейроэндокринной системы, свойственную саркоидозу.

Гормональные нарушения в 90% случаев являются необратимыми, в связи с этим пациенты нуждаются в непрерывной пожизненной заместительной терапии [5][13]. У представленного пациента сохранились явления пангипопитуитаризма инесахарного диабета после регресса саркоидной гранулемы, что свидетельствует о сохранении гормональных нарушений в данном случае. Персистирующие неспецифические симптомы на фоне компенсации нейроэндокринных расстройств явились признаком сопутствующего тяжелого заболевания.

## ЗАКЛЮЧЕНИЕ

Представленный клинический случай интересен нехарактерным для генерализованного саркоидоза дебютом заболевания. Первыми клиническими проявлениями стали симптомы гиперпролактинемического гипогонадизма, к которым последовательно присоединились проявления вторичного гипотиреоза, надпочечниковой недостаточности и несахарного диабета. Длительное время пациент наблюдался по поводу нейроэндокринных расстройств. Однако, несмотря на достижение биохимической компенсации гиперпролактинемии, гипопитуитаризма и несахарного диабета, значительного улучшения в состоянии пациента не наступало.

В ходе динамического наблюдения было установлено, что причиной прогрессирующего ухудшения общего самочувствия было поражение гипоталамо-гипофизарной области в рамках генерализованного саркоидоза. Только после начала его лечения удалось достичь выраженной положительной динамики самочувствия.

У пациентов с такими нейроэндокринными расстройствами, как гиперпролактинемия, гипопитуитаризм и центральный несахарный диабет, необходимо ­оценивать состояние не только гипофиза, но и гипоталамуса. При наличии каких-либо образований в гипоталамической области следует принимать во внимание возможность мультисистемного патологического процесса, в частности — саркоидоза, что определяет тактику дальнейшего обследования пациента.

## ДОПОЛНИТЕЛЬНАЯ ИНФОРМАЦИЯ

Источники финансирования. Работа выполнена по инициативе авторов без привлечения финансирования

Конфликт интересов. Авторы декларируют отсутствие явных и потенциальных конфликтов интересов, связанных с содержанием настоящей статьи.

Участие авторов. Все авторы одобрили финальную версию статьи перед публикацией, выразили согласие нести ответственность за все аспекты работы, подразумевающую надлежащее изучение и решение вопросов, связанных с точностью или добросовестностью любой части работы.

Согласие пациента. Пациент добровольно подписал информированное согласие на публикацию персональной медицинской информации в обезличенной форме в журнале «Проблемы эндокринологии».
